# Secondary Metabolites of a Mangrove Endophytic Fungus *Aspergillus terreus* (No. GX7-3B) from the South China Sea

**DOI:** 10.3390/md11072616

**Published:** 2013-07-19

**Authors:** Chun-Mei Deng, Shi-Xin Liu, Cai-Huan Huang, Ji-Yan Pang, Yong-Cheng Lin

**Affiliations:** 1 School of Chemistry and Chemical Engineering, Sun Yat-sen University, Guangzhou 510275, China; E-Mails: dcm2382405@yahoo.cn (C.-M.D.); lsxinhoho@gmail.com (S.-X.L.); 2 College of Science, Guangdong Ocean University, Zhanjiang 524088, China; 3 Guangdong Province Key Laboratory of Functional Molecules in Oceanic Microorganism, Bureau of Education, Sun Yat-sen University, Guangzhou 510080, China; 4 School of Science and Engineering, Jinan University, Guangzhou 510632, China; E-Mail: caihuan2@sina.com.cn

**Keywords:** mangrove endophytic fungi, thiophene, secondary metabolites, cytotoxicity, AChE

## Abstract

The mangrove endophytic fungus *Aspergillus terreus* (No. GX7-3B) was cultivated in potato dextrose liquid medium, and one rare thiophene compound (**1)**, together with anhydrojavanicin (**2**), 8-*O*-methylbostrycoidin (**3**), 8-*O*-methyljavanicin (**4**), botryosphaerone D (**5**), 6-ethyl-5-hydroxy-3,7-dimethoxynaphthoquinone (**6**), 3β,5α-dihydroxy-(22*E*,24*R*)-ergosta-7,22-dien-6-one (**7**), 3β,5α,14α-trihydroxy-(22*E*,24*R*)-ergosta-7,22-dien-6-one (**8**), NGA0187 (**9**) and beauvericin (**10**), were isolated. Their structures were elucidated by analysis of spectroscopic data. This is the first report of a natural origin for compound **6**. Moreover, compounds **3**, **4**, **5**, **7**, **8** and **10** were obtained from marine microorganism for the first time. In the bioactive assays *in vitro*, compounds **2**, **3**, **9** and **10** displayed remarkable inhibiting actions against α-acetylcholinesterase (AChE) with IC_50_ values 2.01, 6.71, 1.89, and 3.09 μM, respectively. Furthermore, in the cytotoxicity assays, compounds **7** and **10** exhibited strong or moderate cytotoxic activities against MCF-7, A549, Hela and KB cell lines with IC_50_ values 4.98 and 2.02 (MCF-7), 1.95 and 0.82 (A549), 0.68 and 1.14 (Hela), and 1.50 and 1.10 μM (KB), respectively; compound **8** had weak inhibitory activities against these tumor cell lines; compounds **1**, **2**, **3**, **4**, **5**, **6** and **9** exhibited no inhibitory activities against them.

## 1. Introduction

**Figure 1:**
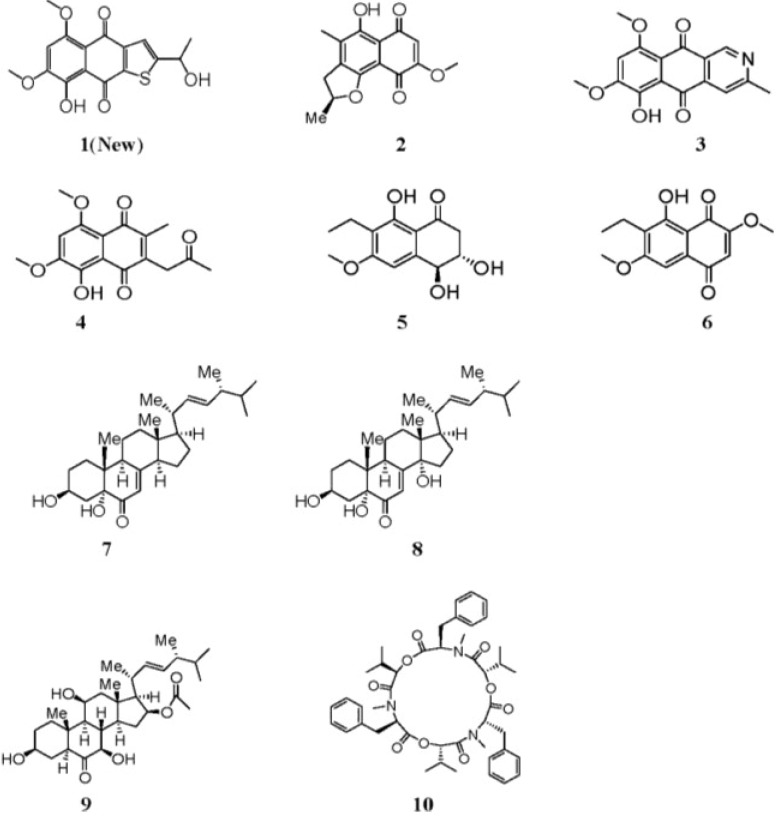
Structures of **1**–**10**.

Marine fungi have been proven to be an important source of structurally novel and bioactive secondary metabolites [1,2,3,4,5], and it is well-known that under various culture conditions, many marine fungi can produce various secondary metabolites, which possess unique structures and bioactivities [6,7]. *Aspergillus terreus* (No. GX7-3B) is a mangrove endophytic fungal strain from the South China Sea. We have previously reported that four sesquiterpenes together with cyclo [IIe–IIe] dipeptide, ergosterol and ergosterol peroxide were isolated from this strain using glucose yeast-extract peptone (GYP) as the cultivation medium. The sesquiterpenes included botryosphaerin F, a new bioactive compound [8]. However, on further investigation, when the fungal strain was fermented on potato dextrose broth (PDB) medium, many different metabolites from those cultivated on GYP medium were obtained, including compound (**1**), as well as anhydrojavanicin (**2**), 8-*O*-methylbostrycoidin (**3**), 8-*O*-methyljavanicin (**4**), botryosphaerone D (**5**), 6-ethyl-5-hydroxy-3,7-dimethoxynaphthoquinone (**6**), 3β,5α-dihydroxy-(22*E*,24*R*)-ergosta-7,22-dien-6-one (**7**), 3β,5α,14α-trihydroxy-(22*E*,24*R*)-ergosta-7,22-dien-6-one (**8**), NGA0187 (**9**) and beauvericin (**10**) (see Figure 1) [9,10,11,12,13,14,15,16,17,18,19]. Compound **1** possessed the naphtho[2,3-*b*]thiophene-4,9-dione system, such a structure hasn’t been encountered in the described yet from nature. This is the first time compound **6** has been isolated from natural sources. In addition, compounds **3**, **4**, **5**, **7**, **8** and **10** were isolated from marine microorganism for the first time. Their inhibitory activities against AChE and their cytotoxic activities against MCF-7, A549, Hela and KB cell lines were examined *in vitro*. Herein, we report the isolation, structure elucidation and biological activities of these compounds.

## 2. Results and Discussion

The crude extract was subjected to a combination of column chromatography on silica gel, sephadex LH-20, C18 reversed phase silica gel, HPLC and reverse phase HPLC.

**Figure 2:**
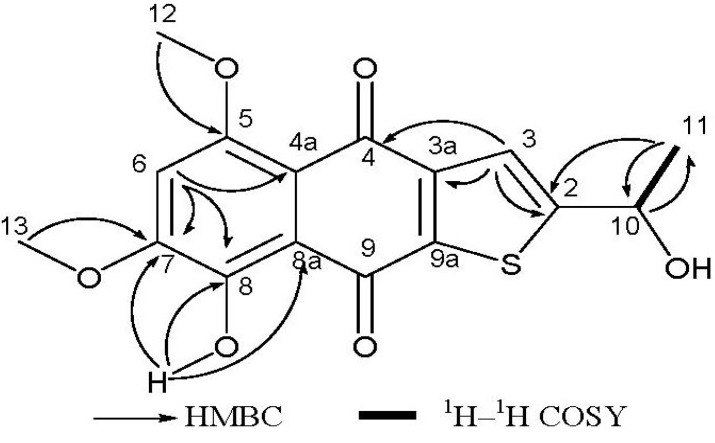
Key ^1^H–^1^H COSY and HMBC correlations for compound **1**.

Compound **1** was isolated as a reddish-orange solid. The HR-EI-MS analysis provided the molecular formula C_16_H_14_O_6_S (obsd *m*/*z* *=* 334.0501 [M]^+^, calcd 334.0506), indicating the presence of one sulfur atom, and requiring 10 degrees of unsaturation. The UV spectrum absorption band at 259 nm suggested a benzene ring chromophore. ^1^H NMR spectrum had showed two hydroxy groups (δ_H_ = 3.49, s, and δ_H_ = 13.53, s), one of which was H-bonded, one methyl group (δ_H_ = 1.71, d, *J* = 6.4 Hz), two *O*-bearing methyl groups (δ_H_ = 4.03 and 4.04, s), two aromatic protons (δ_H_ = 6.84, s and δ_H_ = 8.09, s), one *O*-bearing methine (δ_H_ = 5.65, q, *J* = 6.4 Hz). The ^13^C NMR spectrum showed two carbonyl signals (δ_C_ = 177.5 and 187.3), ten aromatic carbon signals (δ_C_ = 104.2, 112.0, 117.2, 128.2, 129.4, 140.2, 149.6, 155.6, 156.5 and 162.7), accounting for 7 of 10 degrees of unsaturation required by the molecular formula. These data revealed that compound **1** was a three-ring compound, and was probably a naphthoquinone compound (see Table 1). This was supported by comparison with the reported NMR data for naphthoquinones [20]. The sulfur atom should be in the third ring according to the analysis of the chemical shifts of remaining carbons, especially the downfield methine at δ_C_ 65.6, which could only be connected to a hydroxy group and not to a sulfydryl thiol. The 2D NMR spectra (HMBC and COSY) enabled the determination of the overall structure of compound **1** (see Figure 2). The coupled signals from H-10 to H-11 in the ^1^H–^1^H COSY spectrum, and HMBC correlations of H-10 with C-11, of H-11 with C-2 and C-10, and of H-3 with C-2, C-3a and C-4 constructed the contiguous sequence from C-11 to C-4. The hydroxy group (δ_H_ = 3.49, OH) was located at C-10 (δ_C_ = 65.6). So the sulfur atom must be positioned between C-2 and C-9a. The HMBC correlations of H-6 with C-4a, C-7 and C-8, the correlations of H-12 with C-5, of H-13 with C-7, and of 8-OH with C-7, C-8 and C-8a established the positions of two methoxyl groups and downfield hydroxy group. In general, the chemical shift of a H-bonded carbonyl carbon is at lower field than one that isn’t H-bonded, and the assignment of two carbonyl carbons (δ_C_ = 177.4, C-4, δ_C_ = 187.3, C-9) could also be adequately presented by comparison with naphtoquinones data from literature. The amount of compound **1** was very small, so its absolute stereochemistry has not been determined. All data indicated that compound **1** is a rare thiophene compound, named 8-hydroxy-2-[1-hydroxyethyl]-5,7-dimethoxynaphtho[2,3-*b*] thiophene-4,9-dione.

**Table 1:** NMR spectroscopic data of compound **1** ^a^ (in chloroform-d, δ in ppm, *J* in Hz).

No.	δ_C_	δ_H_	^1^H–^1^H Cosy	HMBC (H to C)
2	162.7			
3	128.2	8.09 (1H, s)		C-2, C-3a, C-4
3a	129.4			
4	177.5			
4a	112.0			
5	156.5			
6	104.2	6.84 (1H, s)		C-4a, C-7, C-8
7	155.6			
8	149.6			
8a	117.2			
9	187.3			
9a	140.2			
10	65.6	5.65 (1H, q, *J* = 6.4)	H-11	C-11
11	23.3	1.71 (3H, d, *J* = 6.4)		C-2, C-10
12	57.4	4.03 (3H, s)		C-5
13	56.6	4.04 (3H, s)		C-7
8-OH		13.53 (s)		C-7, C-8, C-8a
10-OH		3.49 (s)		

^a^ Measured at 400 MHz (for ^1^H) and 101 MHz (for ^13^C).

The known compounds were identified as compounds **2**–**10** by spectral analysis and comparison with reported literature data. Isolated of compound **6** from natural sources hasn’t previously been reported, although it has been synthesized [14]. In addition, compounds **3**, **4**, **5**, **7**, **8** and **10** were obtained from marine microorganism for the first time.

All compounds **1**–**10** were evaluated as AChE inhibitors, following the method described by Ellman [21], using Huperzine A as reference. Compounds **2**, **3**, **9** and **10** displayed inhibitory activity of this enzyme with the IC_50_ values 2.01, 6.71, 1.89, and 3.09 μM respectively (see Table 2).

Compounds **1**–**10** were further evaluated for inhibitory activities against human breast cancer cells (MCF-7), lung cancer cells (A549), cervix carcinoma cells (Hela) and human nasopharyngeal carcinoma cells (KB). These cell lines were incubated for 72 h with increasing concentrations of compounds **1**–**10** respectively, and Epirubicin was used as a positive control. IC_50_ of these compounds were determined by the MTT assay [22] (see Table 2). The results showed compounds **7** and **10** exhibited strong or moderate cytotoxic activies against all of MCF-7, A549, Hela, and KB cell lines; compound **8** had weak inhibitory activities against these tumor cell lines; compounds **1**, **2**, **3**, **4**, **5**, **6** and **9** exhibited no inhibitory activities against MCF-7, A549, Hela and KB cells lines.

**Table 2:** The inhibitory activities against AChE and cytototicities towards tumor cell lines of compounds **1**–**10** *in vitro*.

Compounds	Inhibition of AChE	Cytotoxicity			
	IC_50_ (μM)	IC_50_ (μM)			
		Hela	A549	MCF-7	KB	
1	-	-	-	-	-
2	2.01	-	-	-	-
3	6.71	-	-	-	-
4	-	-	-	-	-
5	-	-	-	-	-
6	-	-	-	-	-
7	-	4.98	1.95	0.68	1.50
8	-	25.4	27.1	24.4	19.4
9	1.89	-	-	-	-
10	3.09	2.02	0.82	1.14	1.10
Huperzine A ^a^	0.003				
Epirubicin ^a^		1.07	0.79	0.42	0.05

^a^ as a positive control; “-“ as “no action”.

## 3. Experimental Section

### 3.1. General

Column chromatography (CC) was carried out on silica gel (200–300 mesh, Qingdao marine Chemical) and sephadex LH-20 (GE Healthcare, Pharmacia, Sweden). The HPLC system consisted of a Waters 2010 series. A mini ODS column (250 × 10 mm, 10 μm particle size) was used. Melting point (m.p.) was detected on Fisher-Johns hot-stage apparatus and was uncorrected. Optical rotation was measured on a Schmidt Haensch Polartronic HH W5 polarimeter and was uncorrected. NMR data were recorded in chloroform-d, using TMS as internal reference on a Varian Inova 400 MHz NMR spectrometer (^1^H, 400 MHz; ^13^C, 101MHz). EIMS were on a Thermo DSQ EI-mass spectrometer. LC/MS data were acquired using an Applied Biosystems/MDS Sciex and ESI source. HR-EIMS were measured on a Thermo MAT95XP. UV spectra were measured on a Shimadzu UV-2501 PC spectrophotometer. IR spectra were measured on a Bruker Vector 22 spectrophotometer.

### 3.2. Strain Isolation, Taxonomic Classification and Endophyte Fermentation

The strain of mangrove endophytic fungus *Aspergillus terreus* (No. GX7-3B) was isolated from a branch of *Bruguiera gymnoihiza* (Linn.) Savigny, growing in the coastal salt marsh of the South China Sea in Guangxi province. The strain was stored at School of the Chemistry and Chemical Engineering, Sun Yat-Sen University, Guangzhou, China. It was identified according to a molecular biological protocol by DNA amplification and sequencing of the ITS region as described previously with an ITS sequence GenBank ID: KC 461499. The fungal strain was cultivated on potato dextrose liquid medium (20 g of dextrose and 3 g of crude sea salt in 1 L of potato infusion). Starter cultures were maintained on cornmeal seawater agar. Plugs of agar supporting mycelia growth were cut and transferred aseptically into a 500 mL Erlenmeyer flask containing 200 mL of liquid medium, and incubated at 28 °C on a rotary shaker for 5–7 days. The mycelium was aseptically transferred into 1000 mL Erlenmeyer flasks containing 300 mL PDB medium and incubated at 28 ± 1 °C for 30 days under stationary conditions.

### 3.3. Extraction and Separation of Metabolites

The cultures (130 L) were filtered through cheesecloth. The filtrate was concentrated to about 5 L below 55 °C, and extracted three times with an equal volume of ethyl acetate. The mycelium was air-dried first, and then extracted three times with methanol (5 L × 3). After concentration *in vacuo*, the combined extract (161.9 g) was chromatographed on silica gel CC using gradient elution with petroleum ether (PE) and ethyl acetate (EA) mixture (90:10–0:100, v/v) to give five fractions (1–5). Fraction 2 (15.3 g) was purified by gradient elution with PE-EA mixture (90:10–30:70, v/v) to give compound **4** (11.6 mg), compound **5** (20.4 mg) and compound **6** (15.7 mg). Fraction 3 (10.8 g) was sequentially purified by gradient elution with PE-EA mixture (30:70–50:50, v/v) to give 3-1 (12.6 mg), 3-2 (15.4 mg) and 3-3 (20.1 mg). These three subfractions were further purified by Sephadex LH-20 gel CC with CHCl_3_–MeOH (50:50, v/v) as a mobile phase and by preparative HPLC with an ODS column (10 mm × 250 mm), eluting with MeOH–H_2_O (85:15, v/v) to give compound **2** (2.7 mg), compound **8** (8.2 mg), compound **9** (13.5 mg) and compound **10** (16.5 mg). Fraction 4 (20.7 g) was similarly purified by gradient elution with PE-EA mixture (50:50–70:30, v/v) to give 4-1 (21.6 mg), 4-2 (30.4 mg) and 4-3 (13.7 mg). These three subfractions were further purified by Sephadex LH-20 gel CC with CHCl_3_–MeOH (50:50, v/v) as a mobile phase and by preparative HPLC with an ODS column (10 mm × 250 mm), eluting with MeOH–H_2_O (75:25, v/v) to give compound **1** (2.1 mg), compound **3** (2.4 mg) and compound **7** (5.1 mg).

Compound **1**: Reddish-orange solid. M.p: 131–132 °C. [α]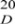 = +271.80 (*c* = 0.883 mg/mL, MeOH). ^1^H NMR (400 MHz, CDCl_3_): δ 1.71 (d, *J* = 6.4 Hz, 3H, H-11), δ 4.03 (s, 3H, H-12), δ 4.04 (s, 3H, H-13), δ 5.65 (q, *J* = 6.4 Hz, 1H, H-10), δ 6.84 (s, 1H, H-6), δ 8.09 (s, 1H, H-3), δ 3.49 (s, OH-10), δ 13.53 (s, OH-8). ^13^C NMR (101 MHz, CDCl_3_): δ 23.3 (C-11), 56.6 (C-13), 57.4 (C-12), 65.6 (C-10), 104.2 (C-6), 112.0 (C-4a), 117.2 (C-8a), 128.2 (C-3), 129.4 (C-3a), 140.2 (C-9a), 149.6 (C-8), 155.6 (C-7), 156.5 (C-5), 162.7 (C-2), 177.5 (C-4), 187.3 (C-9). EI-MS at *m/z* = 334, ESI-MS at *m/z* = 335 [M + H]^+^; HR-EI-MS *m/z*: 334.0501 [M]^+^, (calcd. for C_16_H_14_O_6_S, 334.0506). UV (MeOH): λ_max_ (log ε) = 259 (0.32) nm. IR (KBr): ν_max_ = 3428, 2921, 2852, 1652, 1623, 1544, 1515, 1508, 1460, 1434, 1371, 1316, 1216, 1028, 896, 758, 581, 469 cm^−1^.

### 3.4. Method of *α*-Acetylcholinesterase Inhibitory Activities

α-Acetylcholinesterase inhibitory activity was assayed using 50 mM phosphate buffer (pH 7.0). Compounds **1**–**10** were dissolved in DMSO to different concentrations. 10 μL of sample solution and 10 μL of α-acetylcholinesterase solution (2 units mL^−1^, in the phosphate buffer at pH 7.0) were added to 960 μL of phosphate buffer and incubated at 37 °C for 30 min, and then 20 μL of substrate were added to initiate the enzyme reaction. The enzyme reaction was carried out at 37 °C for 30 min. Product was monitored spectrophotometrically by measuring the absorbance (λ = 400 nm). Huperzine A was used as reference standard inhibitor for comparison. Dose-response curves were obtained by performing assays in the presence of increasing concentrations of inhibitors. IC_50_ value was determined by interpolation of the dose-response curves. For all tests, the inhibition assays were performed in triplicate.

### 3.5. Cytotoxicity Assays

The cytotoxic activities were determined using MCF-7, A549, Hela, KB human tumor cells by the MTT colorimetric method. Cells were harvested during logarithmic growth phase and seeded in 96-well plates at a density of 1 × 104 cells/mL, and cultured at 37 °C in a humidified incubator (5% CO_2_) for 24 h, followed by exposure to various concentrations of compounds tested for 48 h. Subsequently 20 μL of MTT solution (5 mg/mL) were added to each well and mixed, the cells were then incubated for an additional 4 h. Culture supernatant were removed, 150 μL of DMSO were added to each well to fully dissolve the MTT—formazan crystals. Cell growth inhibition was determined by measuring the absorbance (Abs) at λ = 570 nm using a microplate reader and calculated according to the following equation:

Growth inhibition = (1 − OD of treated cells/OD of control cells) × 100%
(1)

The half maximal inhibitory concentrations (IC_50_) were obtained from linear regression analysis of the concentration-response curves plotted for each tested compound (Bliss’s software). Results were expressed as the mean value of triplicate data points.

## 4. Conclusions

This work showed that *Aspergillus terreus* (No. GX7-3B) could produce a rich variety of secondary metabolites, and small changes in the cultivation conditions could completely shift the metabolic profile of this strain. Its metabolites obtained on PDB medium were very different from those of on GYP medium. When the strain was cultivated on PDB medium, one novel sulfur-containing compound, as well as nine known compounds were isolated, which had exhibited antitumor or AChE inhibition activities.

## Supplementary Files

Supplementary File 1 Supplementary Information (PDF, 285 KB)
