# Supplementary material for: Secondary Metabolites of a Mangrove Endophytic Fungus Aspergillus terreus (No. GX7-3B) from the South China Sea
Source: Mar Drugs. 2013 Jul 19;11(7):2616–24. doi: 10.3390/md11072616 (PMC3736441; doi:10.3390/md11072616)

Supplementary Information

Figure S1. ^{1}H, ^{13}C, Dept 135, Dept 90, ^{1}H–^{1}H cosy, HMQC, HMBC of compound **1**.

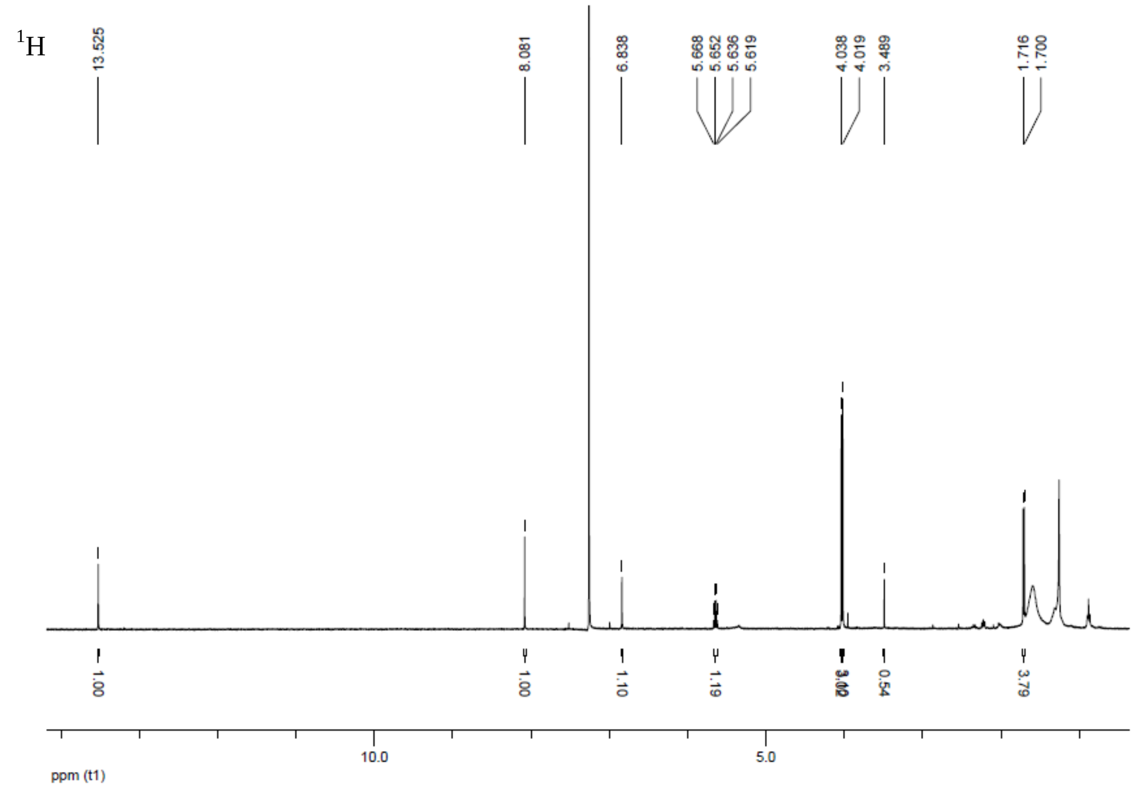

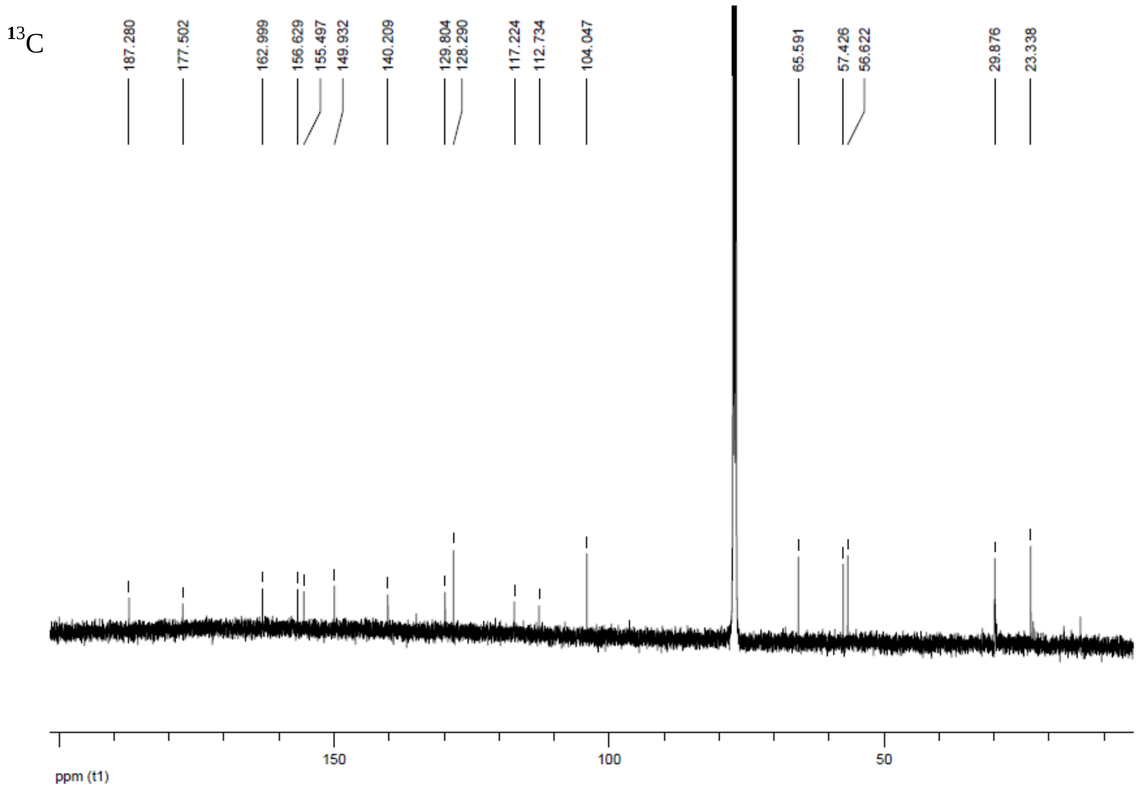

Figure S1. *Cont.*

Dept 135

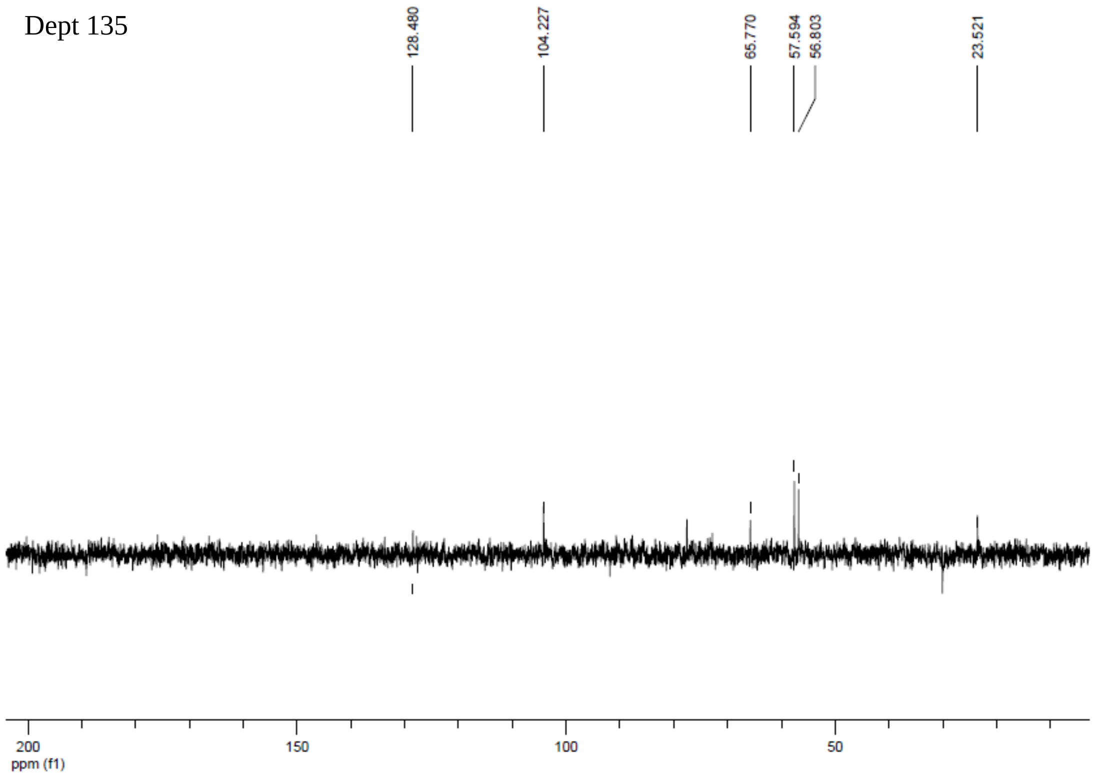

Dept 90

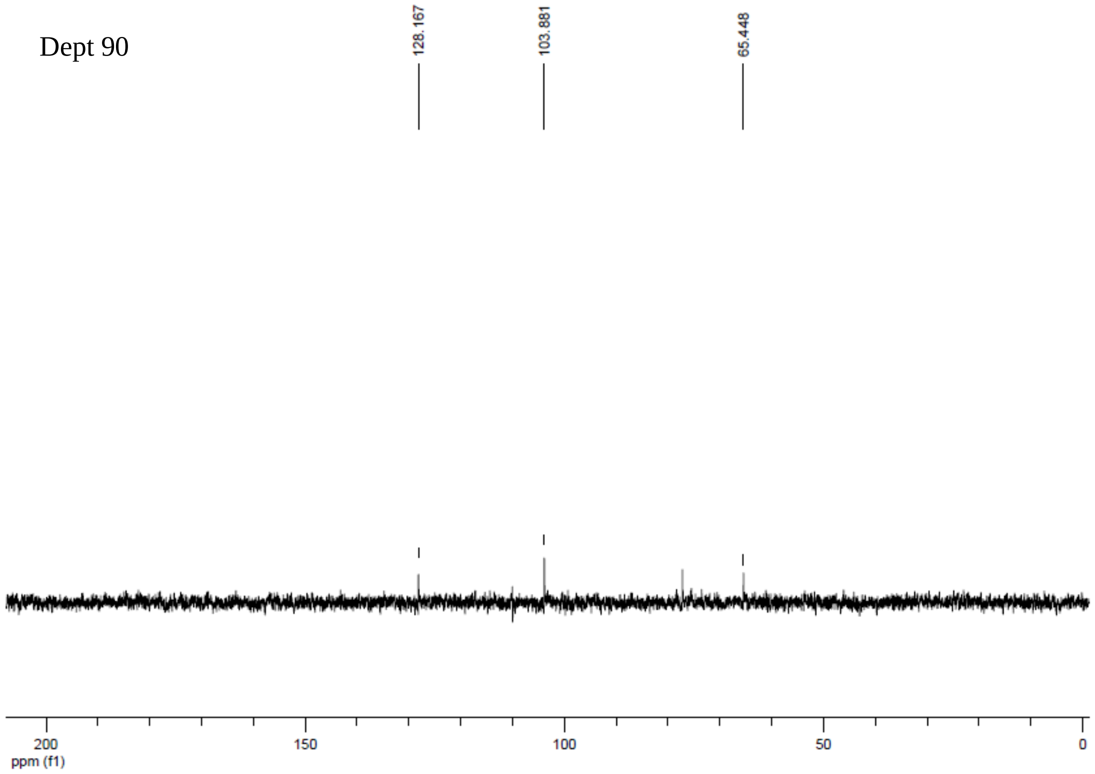

Figure S1. *Cont.*

^1H–^1H COSY

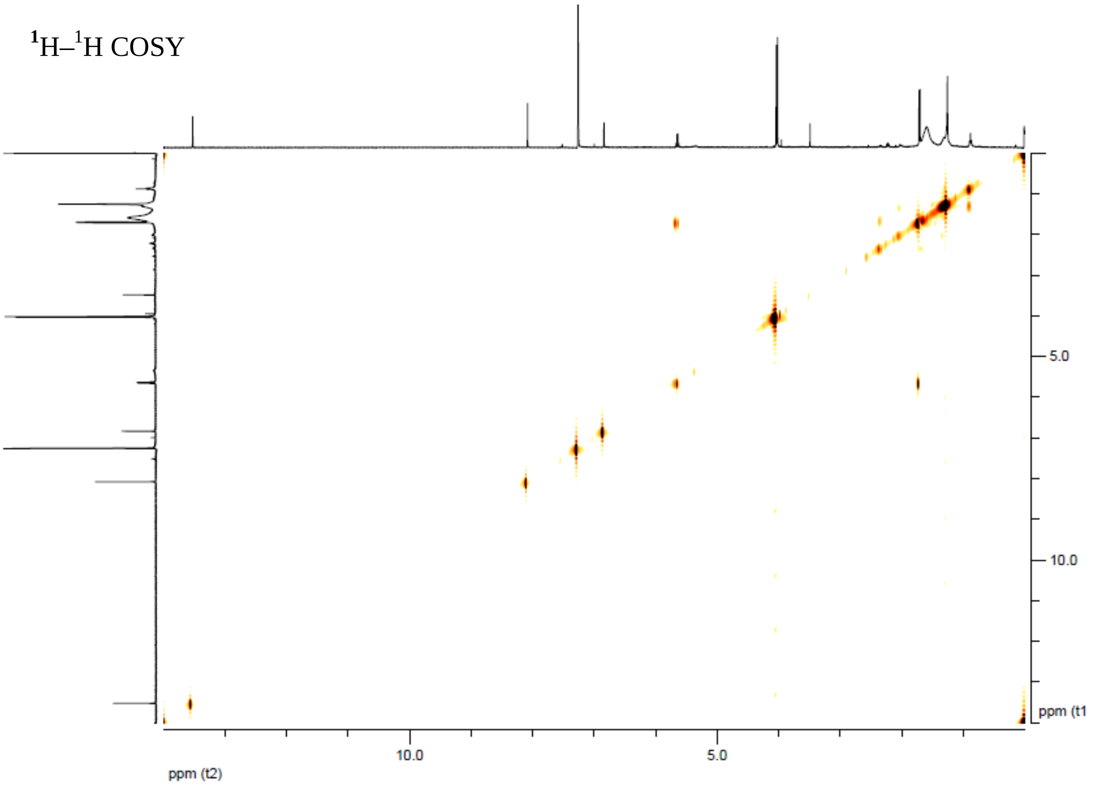

HMQC

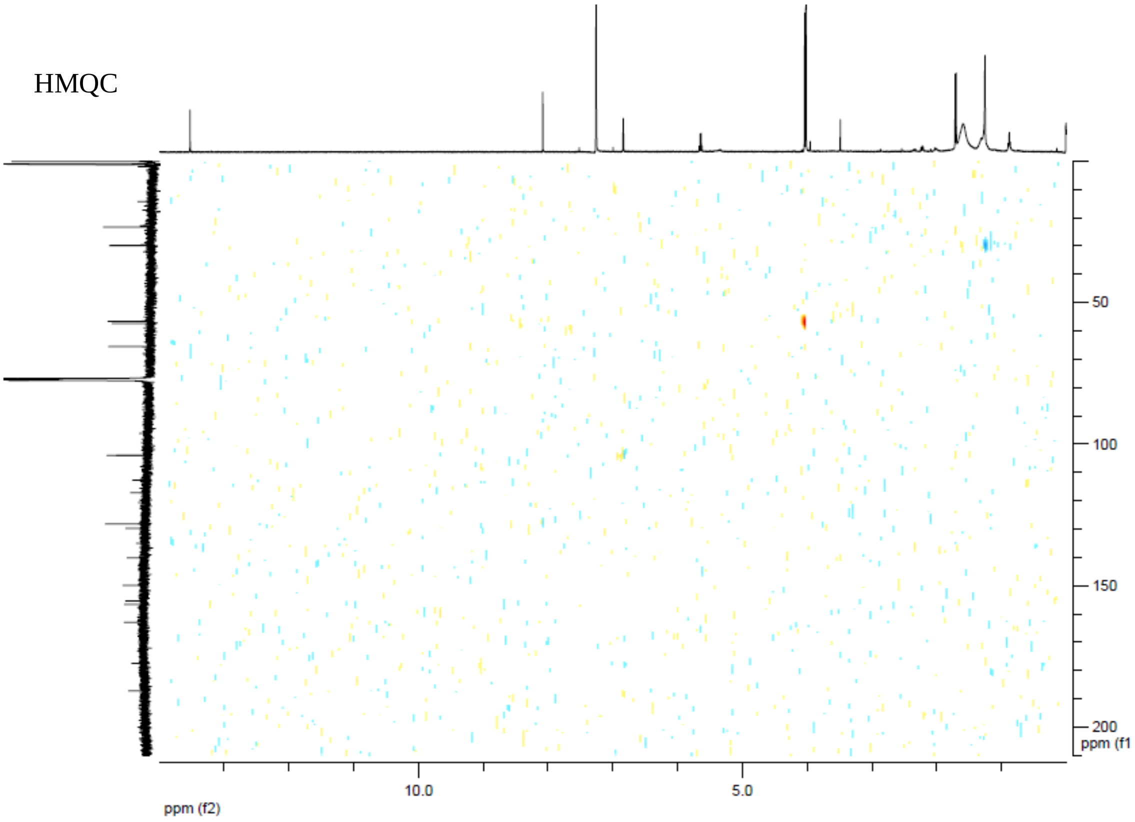

Figure S1. *Cont.*

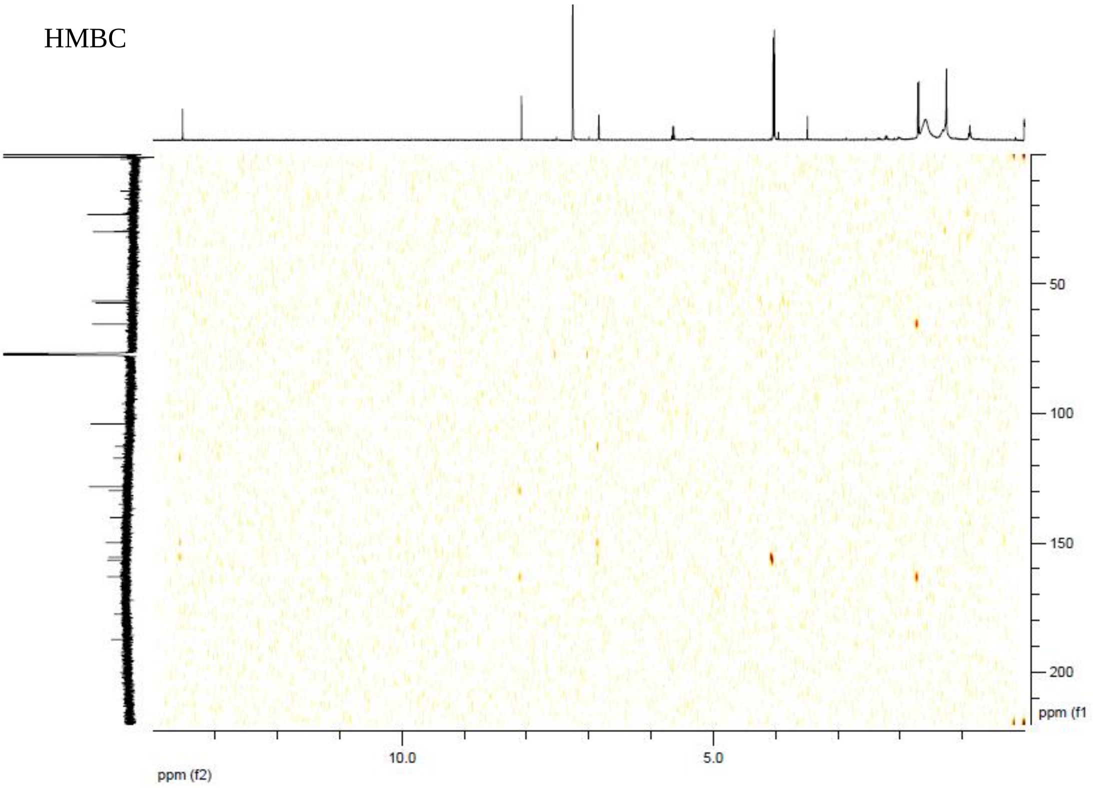

Figure S2. HR-EIMS of compound **1**.

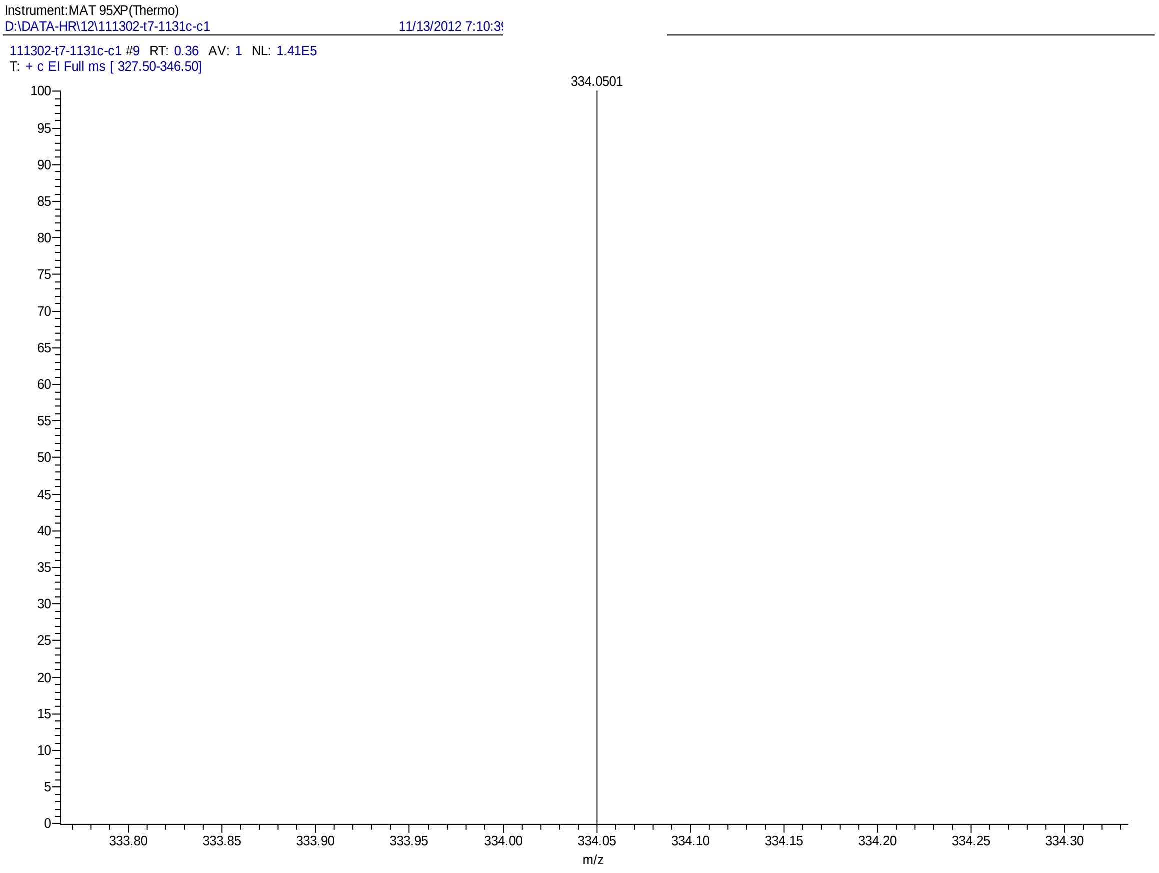

Figure S3. ESI of compound **1**.

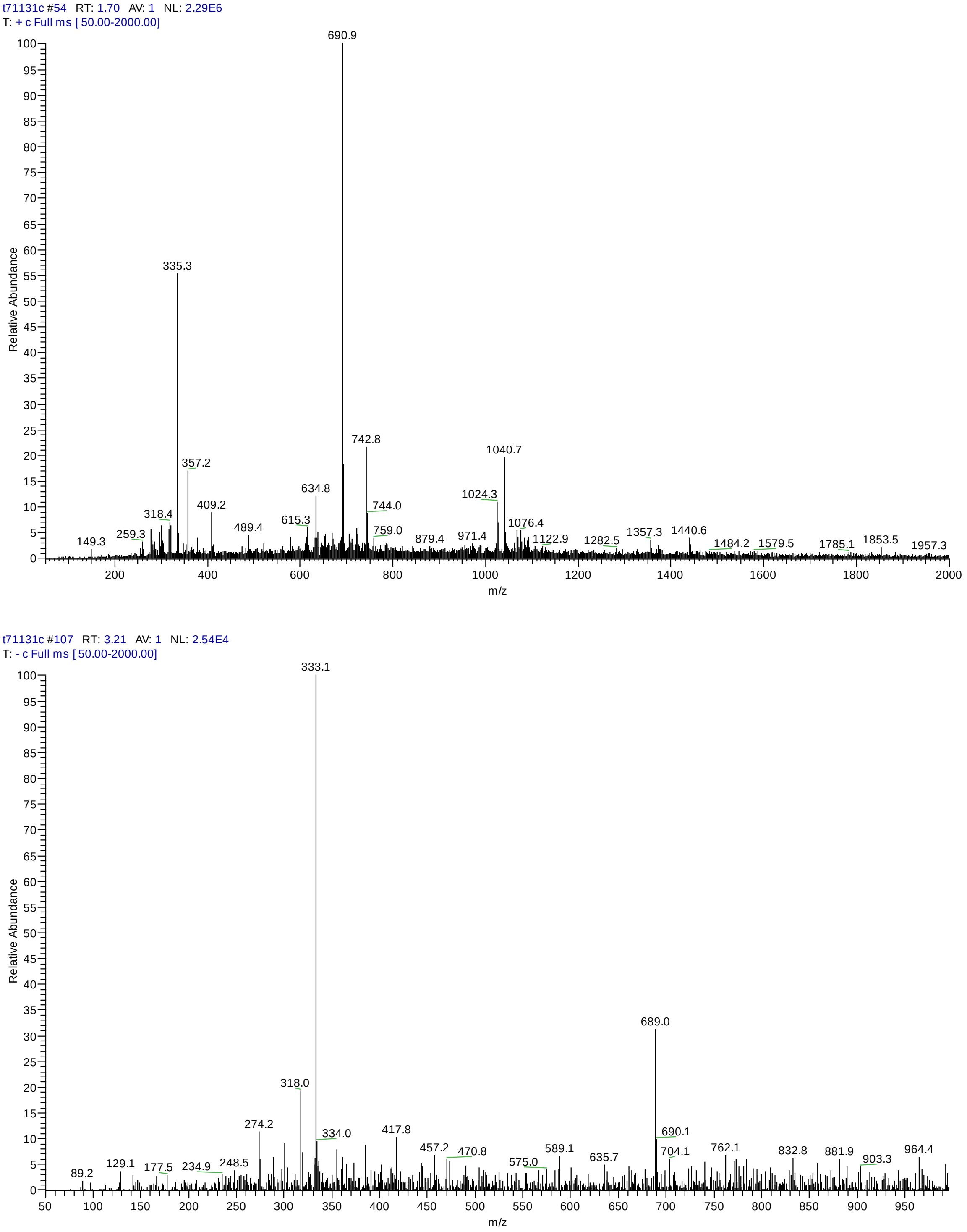

Figure S4. IR of compound **1**.

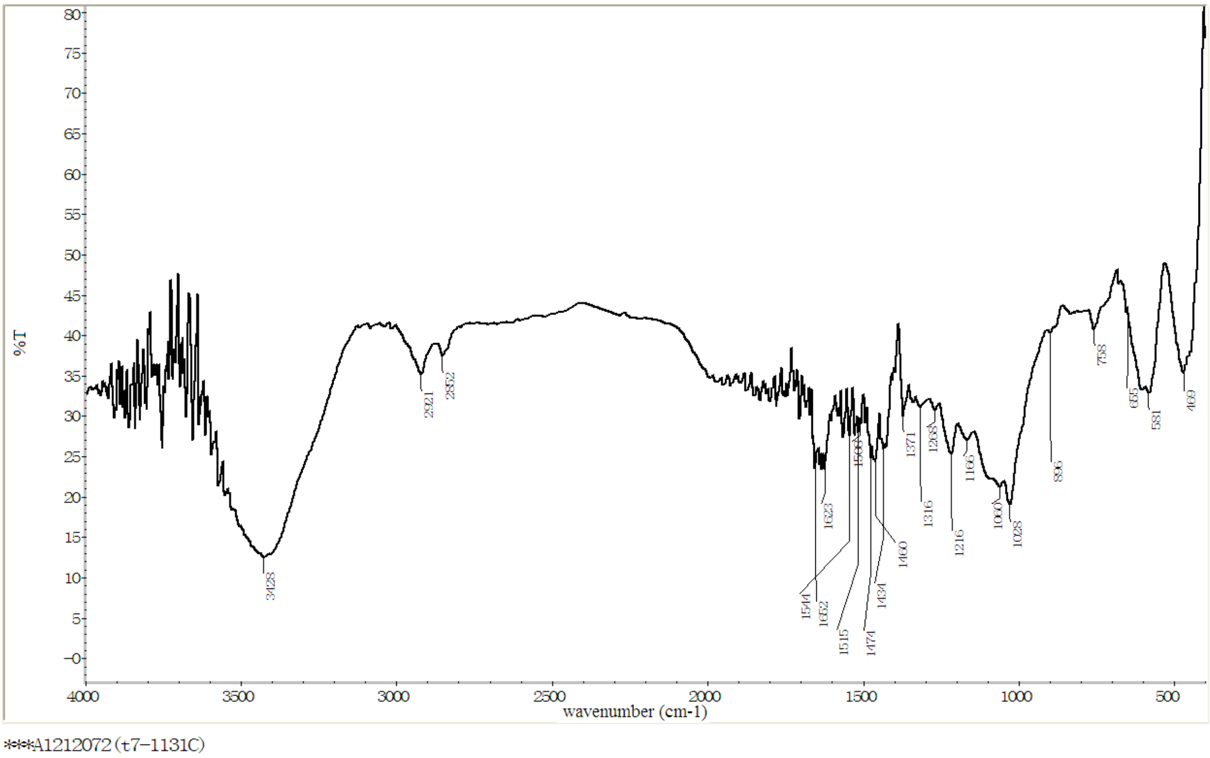

Figure S5. UV of compound **1**.

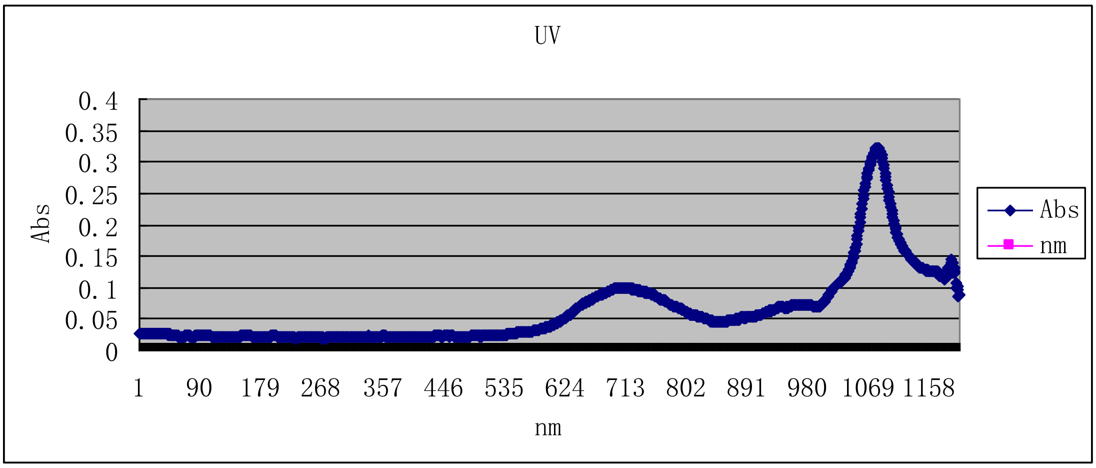

Supplement: Supplementary File 1 — Supplementary Information (PDF, 285 KB) [file marinedrugs-11-02616-s001.pdf]
